# Gentle human interactions trigger positive emotions in chicks

**DOI:** 10.1017/awf.2026.10081

**Published:** 2026-03-30

**Authors:** Javiera Calderón-Amor, Bassam Alhawas, Tamara Tadich, Joanne Edgar, Benjamin Lecorps

**Affiliations:** 1 Universidad Austral de Chile, Chile; 2 Departamento de Ciencias Animales, Facultad de Agronomía y Sistemas Naturales, Pontificia Universidad Católica de Chile, Santiago, Chile; 3 Bristol Veterinary School, United Kingdom; 4 Ciencia Animal, Universidad Austral de Chile, Chile

**Keywords:** Affective state, animal welfare, conditioned place preference, *Gallus gallus domesticus*, gentle handling, human-animal interaction

## Abstract

Interaction with humans in early life is known to influence animal behaviour, stress responses, and welfare, but whether animals perceive gentle handling as emotionally positive remains unclear. Here, we used a conditioned place preference paradigm widely used in affective neuroscience but rarely implemented in poultry, to assess whether chicks experience positive emotions when gently handled. Twenty Hy-Line W-80 chicks were tested in a two-chamber apparatus distinguished by colour cues. Following baseline preference assessment, chicks were exposed to conditioning sessions in which chambers were paired with either gentle handling treatment (soft stroking and calm talking) or a neutral human presence (static and silent). Chicks received six 5-min sessions of each treatment across 12 days, on alternating days (one session per day), and colour-treatment pairings were counterbalanced across individuals. Post-conditioning preference was assessed over three consecutive days. Chicks consistently spent more time in the chamber previously associated with gentle handling across test days. Importantly, chicks did not show an aversion to the neutral chamber. These results indicate that gentle human contact acquired positive associative value rather than merely reducing aversion. These findings provide experimental evidence that human-animal interactions can function as rewarding stimuli in poultry, which has implications for husbandry practices and welfare assessment frameworks.

## Introduction

Human-animal interactions are essential for farm animal welfare (Rault *et al.* 2020; Acharya *et al.* 2022). In chickens, early-life experiences with humans shape behaviour, stress responses, productivity, and overall welfare (Jones 1993; Hemsworth *et al.* 1994; Taylor *et al.* 2022). Unpleasant handling increases fear and reduces productivity (Zulkifli & Nor Azah 2004; Al-Aqil *et al.* 2013; Acharya *et al.* 2022), whereas gentle handling lowers fear and improves welfare (Gross & Siegel 1979; Jones 1993; Taylor *et al.* 2022). For instance, Al-Aqil *et al.* (2013) found that broiler chicks gently handled early in life showed reduced physiological stress responses during transport. In contrast, chicks exposed to unpleasant handling (suspension and noise) showed heightened stress responses and poorer physiological outcomes. However, how much birds value gentle handling and whether these interactions are associated with positive affective states remain unclear.

While most research on avian social motivation has focused on conspecific interactions (e.g. Ferreira *et al.* 2020), the rewarding value of interspecific contact, particularly with humans, remains underexplored in birds. Evidence suggests that even precocial species, such as chicks, preferentially orient toward human-like stimuli shortly after hatching (Rosa-Salva *et al.* 2011). Recent studies have shown consistently that gentle handling can lead to increased voluntary approach and reduced fear towards humans (Calderón-Amor *et al.* 2025b; Stocker *et al.* 2025), suggesting that such interactions are not aversive and may even be rewarding, hinting at cross-species social motivation.

Assessing subjective experiences in animals is challenging, as affective states are not directly observable, and traditional (e.g. behavioural or physiological) measures often capture only transient responses. Animals prefer environments linked to positive or less negative experiences (Kremer *et al.* 2020), and conditioned place preference (CPP) uses these associations to assess how they perceive and remember past events (Nasr *et al.* 2013; Paul *et al.* 2018). For instance, calves prefer locations where they received analgesia after a painful procedure (Ede *et al.* 2019). Unlike conventional methods relying on immediate reactions, CPP assumes affective states shape memories and decision-making (Kirkden & Pajor 2006). Preferences in CPP therefore reflect the learned affective value of prior experiences, with stimuli that are experienced as rewarding typically acquiring positive associative value. It requires minimal training (Hughes *et al.* 1995; Jones *et al.* 2012) and assesses preference without the original stimulus, reducing potential confounding factors (Jones *et al.* 2012). By capturing preferences shaped by past experiences, CPP serves as a powerful tool for studying long-term affective states across species (Millot *et al.* 2014; Ede *et al.* 2024).

Previous studies have shown that chickens can develop conditioned place preferences in response to various environmental cues, such as colour and sound (Jones *et al.* 2012; Ferreira *et al.* 2020). In these studies, chickens were conditioned to associate specific environments, defined by those cues, with rewarding stimuli, such as food, social companionship, or drugs (Hughes *et al.* 1995; Bronson *et al.* 1996; Jones *et al.* 2012; Ferreira *et al.* 2020). However, to our knowledge, no studies have explored whether chickens can develop conditioned preferences in the context of gentle human handling, such as stroking. Hence, this study aimed to determine whether laying hen chicks display a learned preference for environments where they experience gentle handling treatment compared to those with a neutral presence treatment, using a CPP test.

## Materials and methods

### Ethical approval

This study was approved by the Animal Welfare and Ethical Review Body (AWERB) at the University of Bristol (# UIN/24075). All chicks were rehomed as backyard pets at the end of the study.

### Study animals and housing

The study was conducted at the University of Bristol’s Poultry Research Facility with 20 female Hy-line W-80 chicks obtained from a commercial hatchery at 1 day old. The chicks were housed together in a 2.55 × 1.35 m (length × width) indoor pen with 5 cm of wood-shavings, a red feeder and a red drinker providing *ad libitum* access to food (Farmgate Chick Crumbs ACS; ForFarmers UK, Rougham, UK) and water, and two perches. The chicks were provided with two identical electric yellow brooders (EcoGlow Safety 1200; Brinsea Products Itd., Weston-super-Mare, UK) to ensure adequate warmth. Each brooder measured 43.8 × 28.3 cm (width × depth), with an adjustable height of 8–17 cm measured from the base of the brooder. The pen was initially maintained under a 22:2-h light:dark cycle, with light hours gradually decreased to 13.5 h by day 36, following industry-standard, breed-specific management recommendations for Hy-Line W-80 laying hens (Hy-Line International 2022). The pen temperature was initially set at 24°C and reduced to 21°C from day 36 onward. Each chick was marked with a coloured leg ring for identification.

Three people interacted with the animals: two female stockpersons, who were responsible for routine care, and one female experimenter. Stockpersons wore grey overalls and black boots, while the experimenter, responsible for transport and testing, wore a green overall and black boots.

### Conditioned place preference test

#### Apparatus

The experimental apparatus consisted of a rectangular wooden structure measuring 110 × 45 × 40 cm (length × width × height; similar to Jones *et al.* 2012; Nasr *et al.* 2013; Abdulateef *et al.* 2018). It was divided into two equal chambers, each 45 cm in length, separated by a central starting box (20 cm wide). This middle compartment was painted matte white and worked as a starting zone where the tests began. The central box connected to both chambers through movable articulated doors that allowed controlled access to each chamber.

One chamber was orange, and the other blue, as these colours were absent in the home pen and previous research has shown poultry can distinguish these colours (Nasr *et al.* 2013). The front of each chamber (not the starting box) was covered with wire mesh, allowing chicks to see the experimenter while staying inside. Each chamber had a 30 × 30 cm access hatch to allow the experimenter to insert her hands during the gentle handling treatment. During neutral presence treatment, the hatch remained closed; during gentle handling treatment, it was opened to enable human-chick interaction.

**Figure 1.**
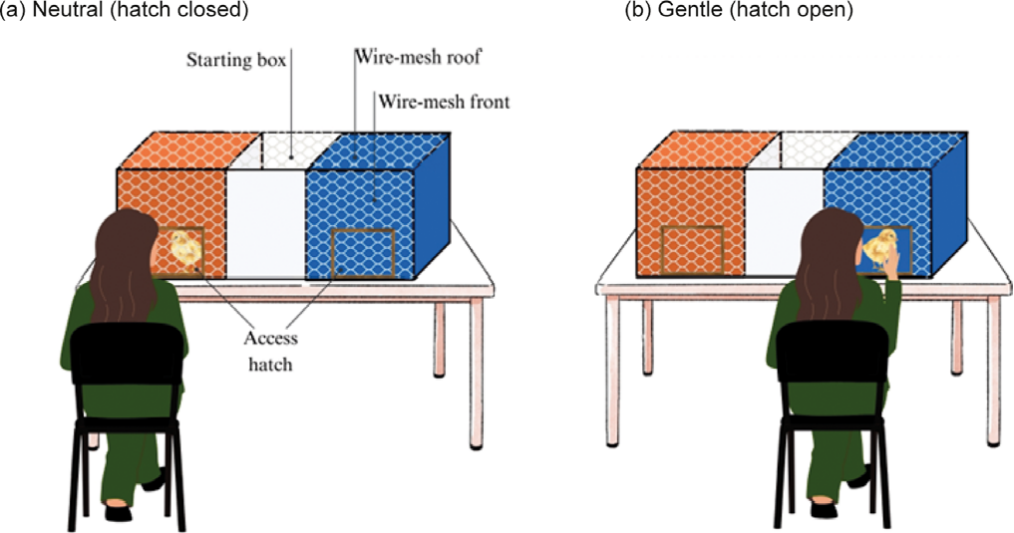
Conditioned place preference apparatus including the neutral human presence (a) and the gentle handling (b). The apparatus featured two chambers with wire-mesh fronts, allowing chicks to see the experimenter. Positioned on a table, the set-up enabled the experimenter to sit in front to conduct conditioning (gentle handling vs neutral presence). Each chamber included a hatch, which was opened during gentle handling and remained closed during neutral presence.

The top of the apparatus was covered with a movable wire-mesh roof, which was opened and closed to place and remove the chicks into the apparatus. The apparatus was placed on a table, and in front of it, the experimenter was able to sit facing each chamber for each session (Figure 1).

#### Procedure

**Figure 2.**
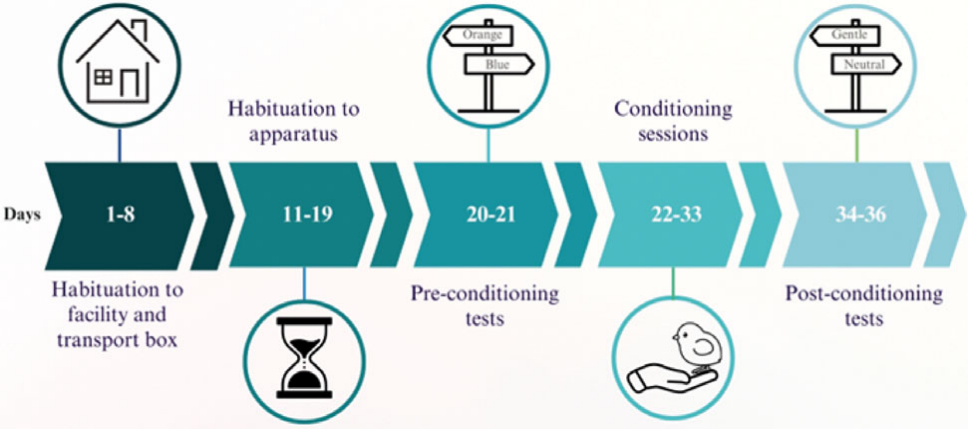
Experimental timeline. Chicks were individually habituated to the facility and handling from days 1–8 and to the testing apparatus from days 11–19. Two pre-conditioning tests were conducted on days 20–21, followed by a conditioning phase from days 22–33. The three post-conditioning tests took place on days 34–36.

The experiment had five phases (Figure 2). Habituation to the housing facilities, habituation to the apparatus, pre-conditioning, conditioning, and post-conditioning were recorded using a GoPro Hero 9 Black (GoPro, Inc., San Mateo, CA, USA) mounted above the apparatus. Pre-conditioning, conditioning and post-conditioning sessions were all done individually.

1. Habituation to the facility and handling (8 days): Chicks were acclimated to the facility and handling. From day 4, the experimenter familiarised them with the transportation cardboard box by placing them one at a time, in groups of five, into the box once per day for 5 minutes before release.
2. Habituation to apparatus (9 days): Chicks were gradually habituated to the experimental apparatus. On the first day, they were introduced in groups of five for two 10-minute sessions (morning and afternoon). For the next six days, habituation was conducted in pairs during a single 10-minute morning session. In the final two days, individual habituation sessions lasted 5 minutes per chamber, also conducted in the morning. Each session allowed access to only one chamber, alternating chambers daily. Habituation always began in the white starting box, with one door removed to allow access to one chamber. The order of exposure was pseudo-randomised.
3. Baseline Place Preference (Precondition Test) (2 days): Each chick was placed individually in the starting box at the centre of the apparatus and allowed access to explore both chambers for 5 minutes. The time spent in the orange and blue chambers was recorded to assess initial preferences. Since chicks exhibited a clear initial preference for the orange chamber, they were ranked based on their time spent in this chamber across both test days. Half of the chicks with strongest preference for the orange chamber were then randomly assigned to receive gentle handling in the orange chamber, while the other half in the blue chamber, ensuring an equal distribution of initial preferences between treatment groups.
4. Conditioning (12 days): During this phase, one chamber was paired with the gentle handling treatment, while the other with the neutral presence treatment (6 sessions per treatment). Each chick was exposed to one chamber per day for 5 minutes. The sessions were carried out between 0800 to 1200h. The chamber colours (orange and blue) were counterbalanced across treatments: half of the chicks received gentle handling in the orange chamber and neutral presence in the blue chamber, and *vice versa*. The order of exposure was consistent throughout the experiment. For both treatments, the experimenter placed the chick into the chamber from the top at the back of the apparatus and waited 10 seconds before sitting in front of the chamber for 5 minutes. During gentle handling, the experimenter opened the access hatch, introduced her hands using slow and deliberate movements, maintained her head at the level of the chick, attempted visual contact, stroked the chick, and spoke softly using positive words in Spanish. In contrast, during neutral presence treatment, the hatch remained closed, and the experimenter stayed still, facing down, avoiding visual interaction. All sessions were video recorded to assess behavioural and vocal responses. Chicks were classified as accepting or avoiding human handling during each session. Acceptance was defined as the chick remaining still and tolerating all stroking attempts without active withdrawal. Avoidance was recorded when the chick moved away during most contact attempts. In cases of avoidance, the experimenter paused briefly (10 s) before gently attempting to resume handling. While it was not possible to fully distinguish freezing from acceptance based solely on posture, acceptance was inferred from the chick’s consistent tolerance to multiple stroking attempts within the session. Additionally, sleeping during handling was recorded. Vocalisations were quantified as the total number of calls per session.
5. Post-Conditioning Test (3 days): The day after conditioning finished, chicks were allowed to roam freely between the chambers to assess changes in their preferences. As in the pre-conditioning tests, each chick was placed in the starting box at the centre of the apparatus and given 5 minutes access to explore both chambers. The experimenter was absent during the test and the hatches were closed. The behaviours recorded included time spent in each chamber and first choice. Chick behaviour was assessed for the pre and post conditioning tests by an observer blind to the experimental treatments using BORIS software.

### Statistical analysis

#### Pre-conditioning tests

During pre-conditioning tests, the time spent in each chamber (orange vs blue) was analysed to assess unconditioned preferences. To compare the time spent in each chamber across both pre-conditioning days, a paired *t*-test was performed using the mean time spent per chick.

Additionally, to evaluate whether chicks entered the orange or blue chamber first more often than would be expected by chance, a binomial test was conducted. This allowed us to determine whether the proportion of chicks selecting one of the two chambers as their first choice was significantly different from the expected 50% chance level.

#### Conditioning sessions

To explore whether chicks became increasingly comfortable with human handling over gentle conditioning sessions, we analysed whether the likelihood of accepting strokes and falling asleep increased across sessions. Two separate generalised linear mixed models with a binomial distribution were used: one with sleeping behaviour (yes/no); and another with accepting strokes (yes/no), as the response variables. In both models, session number was included as a continuous fixed effect to evaluate the cumulative effect of gentle handling, and chick identity was included as a random effect.

To investigate whether the number of vocalisations differed between gentle handling and neutral presence conditioning sessions and how they changed over time, we analysed vocalisations per session using a generalised linear mixed model with a Poisson distribution. Fixed effects included treatment (gentle or neutral), session (as a continuous variable), and their interaction to assess whether the rate of vocalisation change differed between treatments. Chick identity was included as a random effect.

#### Post-conditioning tests

The primary objective was to determine whether treatment (gentle handling vs neutral presence) influenced the time chicks spent in each chamber during the post-conditioning tests. Additionally, extinction learning was examined by analysing chick preference across the three post-conditioning test days.

The response variable was calculated as the percentage of time spent in each chamber during the 300-s post-conditioning test, adjusted by subtracting the average percentage of time spent in the same chamber across the two pre-conditioning tests. This baseline correction was applied individually for each chick and chamber (orange and blue), allowing us to assess changes in chamber preference relative to each chick’s own initial bias. One chick remained in the starting box for the entire 5 min during the first and second post-conditioning tests and was therefore excluded from the analysis (total; n = 19 chicks).

A linear mixed-effects model was used to analyse the effects of treatment (gentle handling vs neutral presence), chamber colour (orange vs blue), test day (1, 2, and 3), their interactions, and test order on the percentage of time spent in each chamber. Chick was included as a random effect.

We conducted *post hoc* analyses to determine whether the observed treatment effect was driven by increased attraction to the gentle chamber or by avoidance of the neutral chamber. Separate linear models were run for each chamber and each post-conditioning day, testing whether the change in time spent (post-conditioning minus pre-conditioning) significantly differed from zero.

Finally, to assess whether chicks were more likely to enter the gentle or neutral chamber as their first choice than expected by chance (different from 50%), we conducted a binomial test separately for each test day.

All analyses were conducted in R (v4.2.2), using the lme4 package (Bates *et al.* 2015) for linear and regression mixed-effects modelling, and ggplot2 (Wickham 2016) for data visualisation. Model assumptions were checked visually and met. Full raw datasets and R scripts used for data processing and statistical analyses are publicly available in Zenodo (Calderón-Amor *et al.* 2025a; https://doi.org/10.5281/zenodo.15306233).

## Results

### Pre-conditioning tests

Chicks showed a strong preference for the orange chamber, based on the mean (± SD) percentage of time spent in each chamber across both pre-conditioning days: 41.38 (± 20.91)% in the orange chamber vs 18.96 (± 12.74)% in the blue chamber; t(30.86) = 3.92; *P* < 0.001). Additionally, on Day 1 of pre-conditioning, 83.33% (15/18) of the chicks chose the orange chamber as their first choice, which was above chance level (binomial test: *P* = 0.003, CI 95%: 60.78–94.16%). However, on Day 2, 52.63% (10/19) of the chicks chose the orange chamber as their first choice, which did not differ from chance (*P* = 0.5, CI 95%: 31.71–72.67%).

### Conditioning sessions

**Figure 3.**
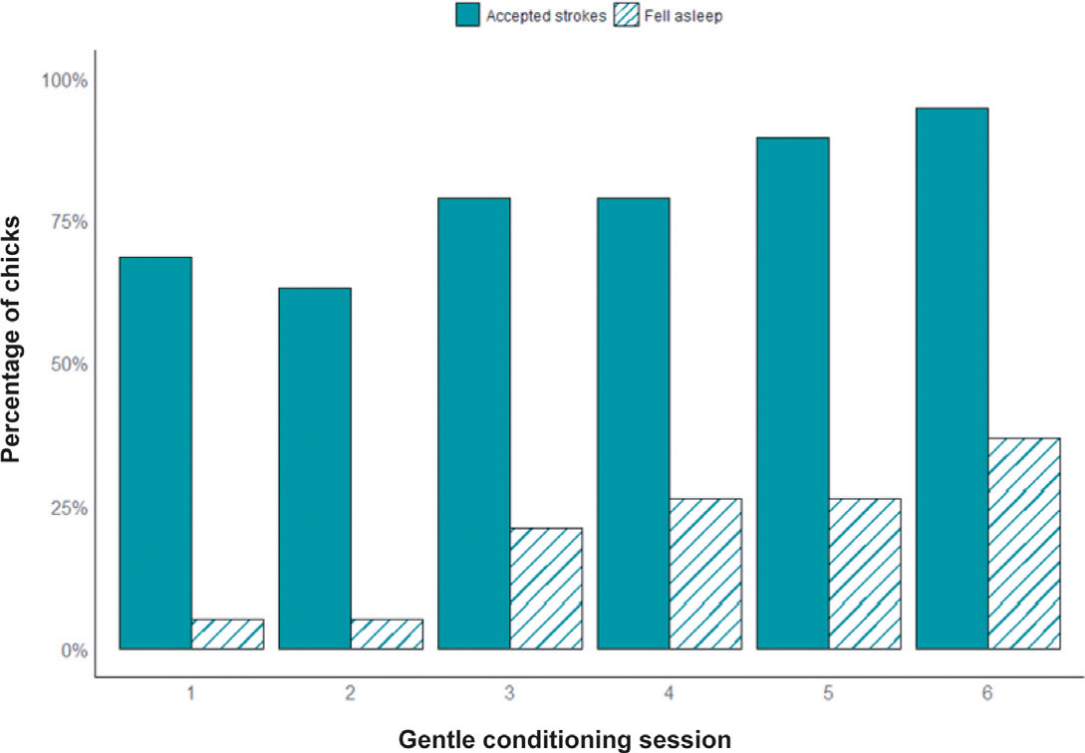
Percentage of chicks (n = 19) that accepted gentle strokes (solid bars) and fell asleep (striped bars) during each of the six gentle conditioning sessions. Bars represent mean values per session.

During gentle sessions, 68% of the chicks (13/19) accepted stroking from the first day without attempting to withdraw. By the last session most chicks (18/19; 95%) accepted the strokes. The likelihood of accepting strokes increased over gentle sessions (OR: 1.73, CI 95%: 1.19–2.53; *P* = 0.004). During the first gentle session, only one chick fell asleep, and by the last session, 47% of the chicks fell asleep at least in one session while receiving gentle handling. The likelihood of chicks falling asleep significantly increased over gentle sessions (OR: 1.64, CI 95%: 1.14–2.36; *P* = 0.007; Figure 3) (Supplementary Material S1).

**Figure 4.**
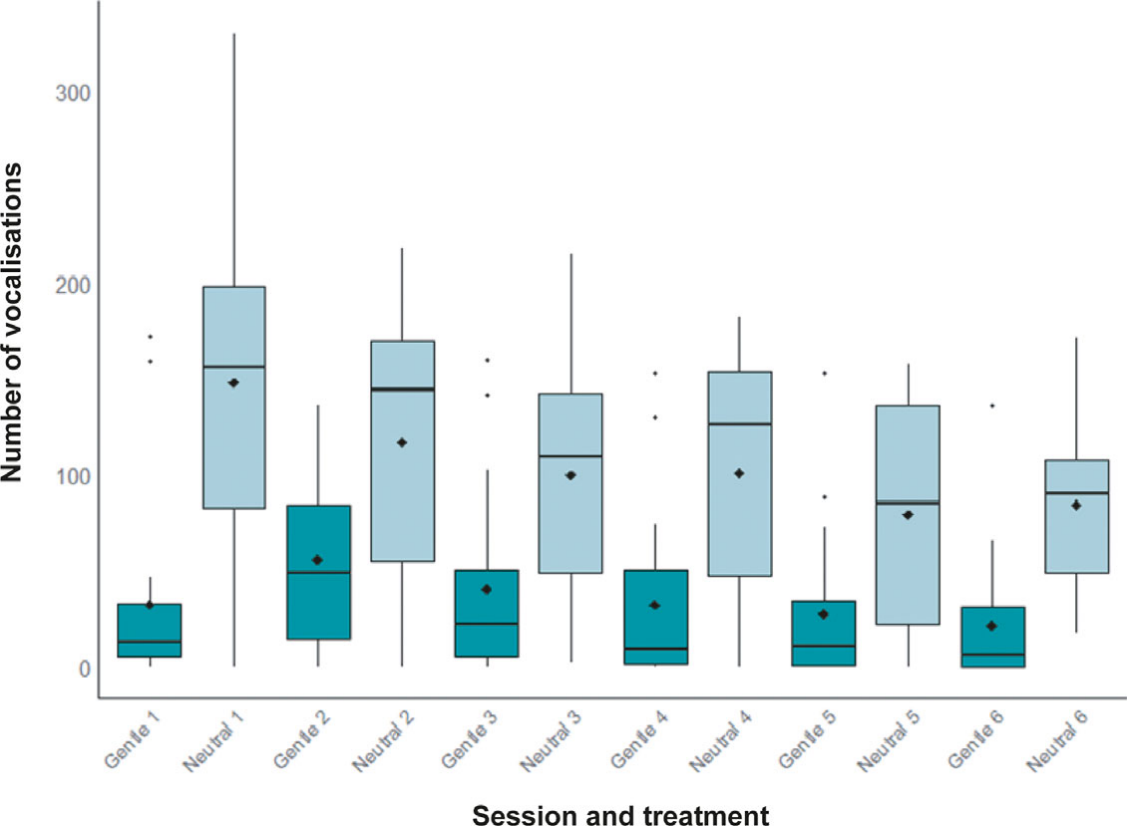
Number of vocalisations recorded during each conditioning session under gentle (dark colour) and neutral (light colour) treatments (n = 19 chicks). Each boxplot shows the interquartile range, the median (horizontal line within the box), and potential outliers. Black diamonds indicate group means.

Chicks vocalised less when in the chamber paired with gentle handling than when in the chamber paired with neutral presence (β = –1.53, SE = 0.04, Z = –34.29; *P* < 0.0001). Vocalisations significantly decreased over sessions, independently of the treatment (β = –0.11, SE = 0.005, Z = –21.98; *P* < 0.0001). The interaction between session and treatment was not significant (β = –0.001, SE = 0.01, Z = –0.14; *P* = 0.88), indicating that the rate of vocalisation reduction across sessions was similar regardless of treatment (Figure 4).

### Post-conditioning test

**Figure 5.**
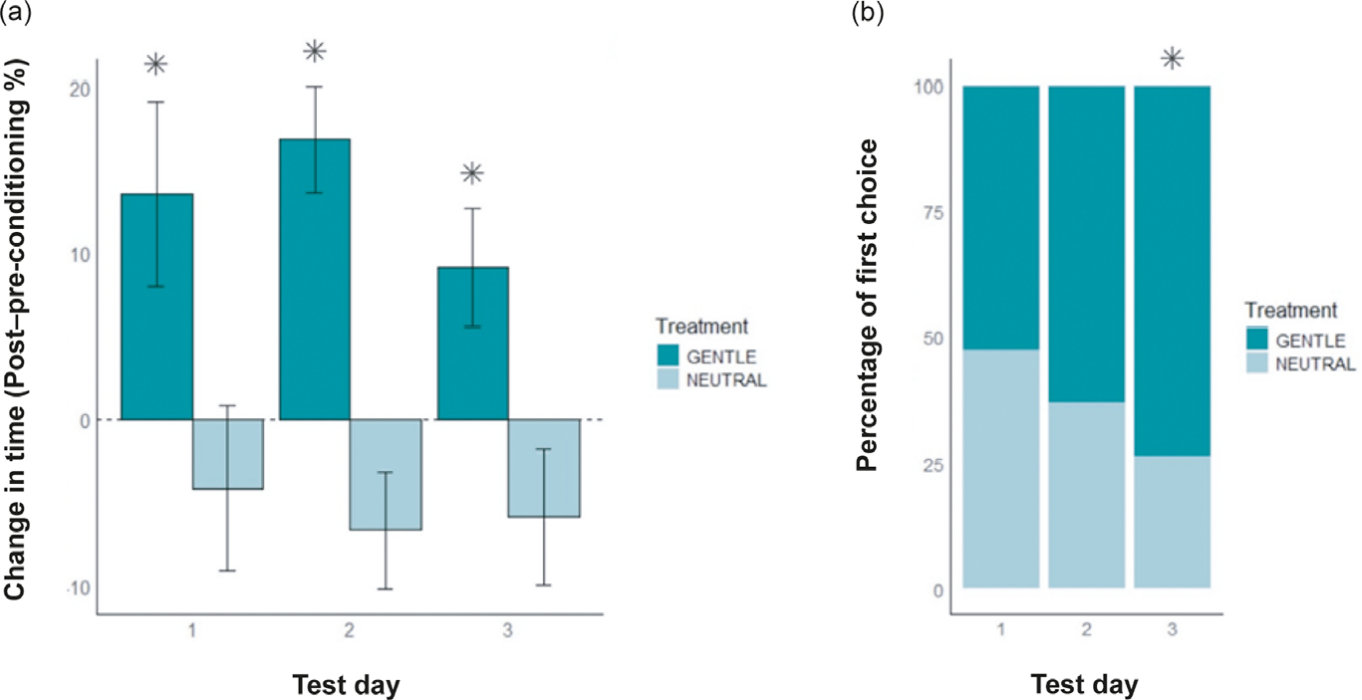
(a) Change in time preference (%) for gentle (dark colour) and neutral (light colour) chambers across the three post-conditioning test days (n = 19 chicks). Bars represent the mean (± SE) percentage change. The dashed line at 0% indicates no change in preference compared to the pre-conditioning phase. (b) Stacked bar chart showing the percentage of chicks selecting the gentle (dark colour) or neutral (light colour) chamber as their first choice across the three post-conditioning test days (n = 19 chicks). Asterisks indicate values significantly different from chance (binomial test; *P* < 0.05).

Across all post-conditioning test days, chicks consistently spent more time in the gentle handling chamber, corresponding to a 16.54% increase relative to pre-conditioning (Estimate = 16.54, SE = 6.95, t(105) = 2.38; *P* = 0.01). Chamber colour and order had no effect, and there was no significant variation across test days (*P* > 0.05; Figure 5[a]).

*Post hoc* analyses focusing on the gentle handling chamber revealed that chicks spent significantly more time in this chamber relative to their pre-conditioning baseline across all three test days: Day 1 (Estimate = 13.55, SE = 5.59, t(18) = 2.42; *P* = 0.02), Day 2 (Estimate = 16.84, SE = 3.27, t(18) = 5.15; *P* < 0.0001), and Day 3 (Estimate = 9.19, SE = 3.57, t(18) = 2.57; *P* = 0.01).

In the neutral chamber, chicks did not show significant changes in time spent across test days. On Day 1 (Estimate = –4.17, SE = 4.98, t(18) = –0.84; *P* = 0.41), Day 2 (Estimate = –6.67, SE = 3.53, t(18) = –1.89; *P* = 0.08), and Day 3 (Estimate = –5.86, SE = 4.05, t(18) = –1.45; *P* = 0.16), time spent remained statistically similar to baseline.

On Day 1 of post-conditioning, 52.63% (10/19) of the chicks chose the gentle handling chamber as their first choice, which did not differ from chance level (binomial test: *P* = 0.5, 95% CI: 31.71–72.67%). On Day 2, 63.16% (12/19) selected the gentle handling chamber, but this result was still not significantly different from chance (*P* = 0.18, 95% CI: 41.09–80.85%). However, on Day 3, 73.68% (14/19) of the chicks chose the gentle handling chamber as their first choice, which was significantly above chance level (*P* = 0.032, 95% CI: 51.21–88.19%) (Figure 5[b]).

## Discussion

In this study, chicks preferred the chamber paired with gentle handling, and this preference persisted across all three post-conditioning test days, even in the absence of gentle interactions. These results suggest that chicks formed a learned association with the chamber paired with gentle handling, which is consistent with a more positively valenced affective response.

The CPP paradigm is a validated method to assess affective states (Kirkden & Pajor 2006; Nasr *et al.* 2013). Previous research has shown that animals display learnt preferences for environments where they experienced positive emotions triggered by drug consumption (e.g. opioid analgesic; Nasr *et al.* 2013) and food reward (Jones *et al.* 2012; Ferreira *et al.* 2020). Our findings align with this evidence, supporting the interpretation that gentle human handling may have induced positive affective states.

Notably, this preference did not diminish, showing no extinction across the three post-conditioning sessions. In CPP paradigms, extinction across repeated test sessions is commonly observed, as the conditioned stimulus is no longer present during testing and each exposure may progressively weaken the learned association. For instance, Ferreira *et al.* (2020) showed that food-conditioned place preference was evident on the first post-conditioning day but was progressively lost across subsequent extinction days (days 2–6). By contrast, Jones *et al.* (2012) reported that food-paired preferences in chicks can be maintained across post-conditioning sessions. Although three post-conditioning days represent a short time window, the persistence of preference across all sessions in the present study suggests that the learned association was resistant to extinction.

Results in post-conditioning tests are consistent with behaviours expressed in the conditioning phase. Over sessions, chicks progressively increased their acceptance of gentle handling and by the final sessions, many even fell asleep in the experimenter’s hands. These behavioural responses — relaxation, proximity, and sleep — are consistent with indicators of a positive human-animal relationship (Rault *et al.* 2020). Vocalisations, a known indicator of distress in socially isolated chicks (Marx *et al.* 2001), were consistently lower in the gentle treatment, suggesting a soothing effect of gentle handling. While overall calling declined over time in both treatments (likely due to habituation) the persistently lower number of vocalisations in the gentle handling treatment suggests not merely reduced arousal, but a shift in emotional valence. In addition, given that chicks were tested individually, the experimenter may have partially substituted for the absence of conspecifics, raising the possibility that gentle handling functioned not only as a rewarding stimulus but also as a form of social buffering — an effect that may be less evident in studies where birds remain within a group (e.g. Jones & Waddington 1992).

While our results suggest that gentle handling treatment elicited a positive affective response in chicks, our design does not allow identification of which sensory cues (tactile, visual, or vocal) were most influential. This was intentional, as we aimed to replicate a rich, realistic human-animal interaction. Previous studies have shown that even static human presence can reduce fear in poultry (Jones 1993; Barnett *et al.* 1994; Calderón-Amor *et al.* 2025b), though Calderón-Amor *et al.* (2025b) found that observing a conspecific being gently handled was more effective at reducing fear and encouraging approach than passive human presence in broilers. However, the benefits of tactile contact vary. For example, Skalná *et al.* (2023) found no effect of gentle tickling on cognitive bias in laying hens. This variability is consistent with theoretical accounts proposing that affective responses depend on how animals interpret stimuli in light of prior experience (Lecorps & Weary 2024). Thus, birds’ emotional responses to human cues may vary according to their previous interactions with humans.

In our study, the clear response to stroking (increased tolerance to tactile contact, greater sleep during gentle handling, reduced vocalisations relative to the neutral presence treatment, and a persistent conditioned place preference) may be due to the chicks’ young age and developmental stage. Early-life interactions may have greater impact due to neural plasticity, social learning, and a lower likelihood of previous negative experiences (Rault *et al.* 2020). Although positive human-animal bonds can also be formed in adult hens (e.g. Graml *et al.* 2008; Bertin *et al.* 2019), interactions occurring early in life may play a key role in shaping long-term responses to humans.

Our findings are consistent with previous work showing the effectiveness of gentle handling in modulating behaviour and production outcomes in poultry. In chicks, Jones and Waddington (1992) reported that gentle daily handling reduced fear responses and increased exploratory behaviour. In adult hens, both Barnett *et al.* (1994) and Bertin *et al.* (2019) demonstrated that repeated, calm interactions (including stroking, static presence, and soft vocalisations) promoted comfort behaviours and physiological benefits, such as reduced corticosterone, improved egg production, and better reproductive outcomes. Similarly, Graml *et al.* (2008) found that gentle interactions, such as walking, talking, feeding, and touching birds improved hens’ responsiveness to humans under commercial free-range conditions, reducing avoidance and increasing voluntary approach behaviours. More recently, Stocker *et al.* (2025) demonstrated that even brief, early-life exposure to visual and voluntary tactile contact with humans can reduce fear-related responses in broilers, further supporting the role of gentle handling in shaping positive human-animal relationships.

Together, these studies reinforce the idea that gentle handling can shape the emotional perception of humans and their associated contexts, with implications for both welfare and productivity. Likewise, our results suggest that not only conspecifics, but potentially humans, may acquire positive associative value during the early development of chicks. This has implications for understanding emotional plasticity in human-chicken interactions and for designing welfare interventions that account for the potential rewarding nature of positive human contact.

### Study limitations

A limitation of our study is the use of only female chicks, which prevents assessment of potential sex differences in responses to human handling (Rubene & Løvlie 2021). Future studies should also examine the influence of individual personality traits, which may influence behavioural responses and explain some of the variability in response to the human. In addition, a challenge with CPP tests is to determine whether the preference observed is due to gentle handling being positive or to the control treatment being aversive (Ede *et al.* 2019). Chicks spent more time in the gentle handling chamber for all three sessions but did not spend less time in the neutral chamber. This suggests that the effect was mostly driven by an increase in preference for the environment associated with gentle handling rather than an artefact of chicks avoiding the neutral chamber. Future work should explore additional control conditions, such as empty chambers or non-social stimuli, to better isolate the rewarding properties of gentle human handling.

### Animal welfare implications

Our findings suggest that gentle, predictable human contact in early life may be experienced positively by chicks. Although further research is needed to confirm whether similar effects occur under commercial conditions and in group settings these results support the idea that calm and consistent human-animal interactions could help not only to mitigate fear of humans but also to generate positive animal welfare in poultry during routine husbandry. Incorporating simple gentle-handling practices may therefore complement existing management strategies aimed at improving welfare in young birds.

## Conclusion

Our results indicate that chicks developed a conditioned preference for the chamber associated with gentle human handling. This consistent preference suggests that the interaction was affectively salient and experienced more positively than the neutral human presence. The persistence of the preference across test days points to a stable learned association. Taken together, these findings indicate that early-life exposure to gentle human contact can influence chicks’ behavioural responses in ways consistent with more positive affective valence, highlighting the potential value of gentle handling for improving animal welfare.

## Supporting information

10.1017/awf.2026.10081.sm001 Calderón-Amor et al. supplementary movie Calderón-Amor et al. supplementary material

## Data Availability

All data and code supporting the findings of this study are available at Zenodo: https://doi.org/10.5281/zenodo.15306233.

## Supplementary material

The supplementary material for this article can be found at http://doi.org/10.1017/awf.2026.10081.
